# Redefining vaccination coverage and timeliness measures using electronic immunization registry data in low- and middle-income countries

**DOI:** 10.1016/j.vaccine.2019.02.017

**Published:** 2019-03-22

**Authors:** Samantha B. Dolan, Emily Carnahan, Jessica C. Shearer, Emily N. Beylerian, Jenny Thompson, Skye S. Gilbert, Laurie Werner, Tove K. Ryman

**Affiliations:** a Dolan Consulting LLC; PATH, Seattle, USA; b PATH, Seattle, USA; c Department of Global Health, University of Washington, Seattle, USA; d Bill and Melinda Gates Foundation, Seattle, USA

**Keywords:** Immunization, Vaccine, Measures, Routine health information system, Individual-level data, Electronic immunization registry

## Abstract

Vaccine coverage is routinely used as a performance indicator for immunization programs both at local and global levels. For many national immunization programs, there are challenges with accurately estimating vaccination coverage based on available data sources, however an increasing number of low- and middle-income countries (LMICs) have begun implementing electronic immunization registries to replace health facilities’ paper-based tools and aggregate reporting systems. These systems allow for more efficient capture and use of routinely reported individual-level data that can be used to calculate dose-specific and cohort vaccination coverage, replacing the commonly used aggregate routine health information system data. With these individual-level data immunization programs have the opportunity to redefine performance measures to enhance programmatic decision-making at all levels of the health system. In this commentary, we discuss how measures for assessing vaccination status and program performance can be redefined and recalculated using these data when generated at the health facility level and the implications of the use and availability of electronic individual-level data.

## 1 Introduction

Immunization programs strive to ensure that every child within their target population is adequately protected from vaccine-preventable diseases (VPDs). Improving vaccination coverage is a multifactorial effort that requires keen insights and oversight of both the target population and health system. Vaccine coverage is routinely used as a performance indicator for immunization programs both at local and global levels [1], [2]. In most countries, coverage data come from a combination of routine health information systems (RHIS) and periodic population-based surveys, each approach having strengths and weaknesses in terms of representativeness, data quality, and resources [3], [4], [5], [6], [7], [8], [9]. For many national immunization programs, there are challenges and limitations with accurately estimating vaccination coverage based on these available data sources [10], [11], [12]. Accurate coverage estimates are needed by immunization clinic staff, program supervisors, and national and global policy makers to take programmatic action and track progress towards immunization goals. Given this, global organizations such as Gavi, the Vaccine Alliance and the World Health Organization (WHO) have put out calls to action for countries to improve the quality and use of immunization data, with one proposed approach being the uptake of individual-level electronic health record systems.

An increasing number of low- and middle-income countries (LMICs) have begun implementing individual-level electronic immunization registries (EIRs) to replace health facilities’ paper-based tools and aggregate reporting systems. According to the Pan-American Health Organization (PAHO) and the WHO, an EIR is a “confidential, population-based information system that contains data on vaccine doses administered”, it allows for the monitoring of vaccination coverage by provider, vaccine, dose, age, target group, and geographical area, and facilitates the monitoring of individuals receiving immunization [13], [14]. The United States’ Centers for Disease Control and Prevention, further elaborates that EIRs “collect and consolidate vaccination data from multiple health-care providers” as well as generate reminder and recall notifications [15]. When an EIR has additional functionalities, such as vaccine management or interoperability with other electronic systems, it is considered an immunization information system (IIS). These systems allow for more efficient capture and use of routinely reported individual-level data that can be used to calculate dose-specific and cohort vaccination coverage, replacing the commonly used aggregate RHIS data [16]. Electronic systems enable vaccination data to be collected in a standardized, searchable format, so they can be assessed and aggregated in real-time. These data expand opportunities to assess program performance, from being able to calculate all current RHIS aggregate and survey indicators, in addition to new measures not previously calculated. However, LMICs transitioning from paper to digital systems, and aggregate to individual-level data, may face new challenges with interpreting and using information collected by electronic systems.

By moving away from the use of facility data captured on paper to individual-level data captured electronically, immunization programs have the opportunity to enhance programmatic decision-making at all levels of the health system through more granular analytics and performance measurement. Much work has already been done on the use of individual-level data generated by EIRs in high-income countries, but little has been written on the use and implications of EIRs on performance measurement in LMICs. In this commentary, we discuss major considerations for how measures for assessing vaccination status and program performance can be redefined and recalculated using individual-level data generated at the health facility level. These measures are not new or novel, we simply present them in a context specific to immunization programs transitioning from aggregate measures, often generated by paper-based tools, to individual electronic data collection tools in low-resource settings and their potential limitations [14]. The measures we discuss are included in existing national and regional guidance, however we know that limitations exist with the current quality of EIR data, therefore limiting the utility of some of these measures, and forcing immunization programs to continue relying on other sources of information. We aim to describe what it means for LMICs moving from aggregate to individual data by way of introduction of an EIR in terms of performance measurement.

### 1.1 Historic measurement challenges in LMICs

Measuring immunization program performance in LMICs has historically been hindered by the available data sources and data quality, subsequently limiting the utility of measures used to track progress. Papania et al. previously described the three key characteristics needed for information to be used more effectively by immunization program staff: timeliness, accuracy, and particularity [17]. Timeliness refers to how quickly data are accessible, accuracy refers to the correctness of numerator and denominator data, and particularity refers to the level of specificity or granularity of the data, ideally down to each individual and vaccine dose.

Historically common administrative data sources in LMICs have been paper-based immunization registries, aggregate monthly summaries, and home-based records; the monthly summaries are often entered into an electronic database for reporting purposes. These data sources often lack at least one of the characteristics described above. The impracticality of utilizing the paper-based records to calculate performance measures causes programs to rely on unspecific aggregate measures, that are often inaccurate due to recording errors and suffer from poor completeness and timeliness of reporting [6], [11], [12]. Denominator estimates often come from population projections that are produced infrequently causing the accuracy of these estimates to decrease over time [18]. As the WHO has documented, few immunization programs have the resources to independently produce accurate target population estimates. These administrative data sources are supplemented by national surveys, such as the Demographic and Health Survey, conducted every 3–5 years [5]. Although survey data is often considered more accurate than administrative data, it is untimely, and often also unspecific as estimates are made at regional and national levels, in addition to suffering from selection and information biases [19]. Countries have relied on these data sources for decades, thereby consistently grappling with the limitations of the data for measuring progress. These limitations should decrease as electronic information systems become more widely used.

## 2 Common vaccination measures

Below we define and describe commonly used measures amongst LMICs to provide background on how these measures can be improved using individual-level data from EIRs, recognizing that terminology is often used inconsistently by immunization experts [20].

### 2.1 Vaccination coverage

Traditionally, in many LMICs, vaccination coverage has been calculated from RHIS using the number of vaccines administered to any child seen at the facility in a month as the numerator, divided by the denominator of the facility’s estimated monthly target population (estimated annual births/12). Coverage is often disaggregated and reported by children <1 year and ≥1 year receiving each dose. Coverage is calculated at the facility, district, regional, and national levels on a routine basis, however, national coverage estimates are finalized during an annual joint review process that compares administrative to survey data and takes contextual factors into consideration for the final estimate [4].

### 2.2 Drop-out and lost to follow-up

The terms drop-out, incomplete or partial vaccination, and lost to follow-up are often used interchangeably amongst immunization program staff; for the purposes of the measures discussed here, drop-out (similar to incomplete or partial vaccination) is antigen specific and refers to delays in the administration of subsequent scheduled antigen-doses while lost to follow-up refers to a child who does not return to a facility for any subsequent vaccinations. Immunization programs often use drop-out to assess facility performance in terms of the number of children lost to follow-up, as well as a proxy for health system quality and population demand for vaccines. Traditionally drop-out is estimated by subtracting the number of second or third doses from first doses administered each month for a particular vaccine series and divided by the number of first doses at the health facility level. The number of children lost to follow-up is not typically reported by immunization programs, although it is often tracked by individual facilities.

### 2.3 Dose validity and timeliness

On-time vaccinations are crucial for ensuring children acquire and maintain immunity to VPDs. Vaccination validity is specific to a single dose in a vaccine series and important for determining if a child will acquire adequate levels of immunity following vaccination based on their age. Traditionally timeliness refers to whether a child receives a vaccine within a specific timeframe, where they can receive a dose early, on-time, or late. An up-to-date vaccination schedule is generally based on whether all recommended vaccines were administered before a particular age or point in time [20]. Historically dose validity has not been incorporated into performance measures, except for surveys; for aggregate RHIS data in LMICs, doses are counted if they are administered, regardless of their timing.

## 3 Potential benefits of EIRs to improve measurement

Individual-level electronic immunization data offer numerous opportunities to increase the timeliness, accuracy, and particularity of performance measures. The full extent of potential benefits of these new measures has yet to be explored in low-resource settings or with existing software functionality, but we can hypothesize the added value and implications for immunization programs and national health systems. The availability of individual-level data accessible in real-time provides the opportunity to redefine vaccination coverage and drop-out, and enables the calculation of new measures that are not currently captured in routine reporting such as dose validity and timeliness (as summarized in Table 1). Additionally, leveraging EIR data can address accuracy issues inherent in using aggregate administrative data, such as poor recording practices, along with allowing for new measures to be calculated that can allow programs to more easily act on their data at the right time [21]. Primarily the added value of using these data is that they provide a more granular and timely picture of children susceptible to VPDs by age and antigen at both the individual and population levels. We have summarized the strengths and weaknesses of EIR data compared to aggregate administrative RHIS and survey data in Table 2. Below we illustrate how improved definitions can be used by considering how measures would change amongst a small cohort of children.

**Table 1:** Summary table of vaccine measures.

	Current reporting measure (aggregate RHIS data)	Proposed measure (individual RHIS data) [22], [23], [26]	Value added of the proposed measure over the current measure	Alignment with common indicators [1], [44], [45], [46]	Measurement considerations
**Dose Validity**	Not currently reported	Doses administered on or after scheduled date as per national schedule	Greater insight over VPD susceptibility		Guidance on validity and follow-up on invalid doses needed
**Dose Timeliness**	Not currently reported	Doses administered on or after the recommended time interval since previous dose administered	Can be more accommodating; allowing for children to remain on-time following a delayed vaccination		Length of buffer time for being considered on-time
**Coverage**	Number of children receiving vaccination in a given month/year divided by the annual target population estimate per month from census	Number of children within a given age cohort receiving vaccination on or after the recommended time divided by the total number of children in the age cohort captured by the RHIS	More accurate estimate reported at the individual-level and specific estimates at the facility and community levels, allowing for gaps amongst particular groups to be quickly identified	Gavi*: Number of unique children immunized, coverage of pentavalent 3rd dose and measles 1st dose GVAP**: 90% national coverage of DPT-containing vaccines SDG: Coverage of essential services (including vaccines) Regional VAPs#: 90%[47], [48], [49] or 95%[50], [51], [52] DTP national coverage, 95% MCV1 coverage	Does not capture children not seen by the health system; denominator can be defined using multiple criteria
**Drop-Out**	Percentage difference between two doses in the same series for a given month	Percentage difference between two doses in the same series for a given cohort of children (by age or since a particular time)	More accurate estimate reported at the individual-level	Gavi*: Drop out between first and third doses of pentavalent GVAP**: Drop out between first and third doses of DTP-containing vaccines Regional VAPs#: Drop out rates below 5%[47], [51], [52] or 10%[48] between first and third doses of DPT vaccine	
**Lost to Follow-Up**	Not currently reported. At the facility level, sometimes operationalized as children captured in paper-based patient registers who have not returned for scheduled vaccine doses	A child does not return to a facility for the scheduled dose after a particular time period	Improved ability to do targeted follow-up on individuals and sensitivity for identifying those children who will not return; cleaner denominators		Length of buffer time after the scheduled date for being considered lost to follow-up

* Gavi- Gavi, the Vaccine Alliance

** GVAP- Global Vaccination Action Plan, World Health Organization

# Regional VAPs- Regional Vaccine Action Plans

**Table 2:** Strengths and Weaknesses of Routine Data Sources used by Immunization Programs.

	Strengths	Weaknesses
**Electronic Immunization Registry (EIR) Data**	• Individual-level records that include demographic information • Data are easily accessible electronically • Linkage of records across facilities • Updated daily, potentially • Possible for immunization program managers to use to provide real-time feedback • Improved data quality due to built-in validity checks	• High-maintenance system • Potentially incomplete data, can only make estimates for individuals seen at facilities using EIRs • Requires expertise in data management and analysis
**Aggregate Routine Health Information System (RHIS) Data**	• Low-maintenance recording and reporting system • Potentially includes all vaccinated children • Immunization program managers can act on data • Updated routinely	• Lack of granularity below facility level • Poor data quality • Inaccurate denominator estimates • Lack of record linkage
**Survey Data**	• Improved accuracy of estimates • Representativeness of target population • Demographic information of individuals collected • Potentially high quality data	• Untimely estimates • Estimates made only down to sub-national levels • Little use for immunization program managers • Data not easily accessible

### 3.1 Dose validity and timeliness

Unlike paper-based systems, EIRs allow for efficient collection and access of children’s vaccination administration dates and date of birth. This allows for vaccination validity and timeliness to be calculated based on a child’s age and time between administration of doses in a series. Many countries already have specific definitions of validity and timeliness for each recommended vaccine, we simply present some considerations for how countries newly implementing EIRs can calculate these measures [22], [23].

**Fig. 1:**
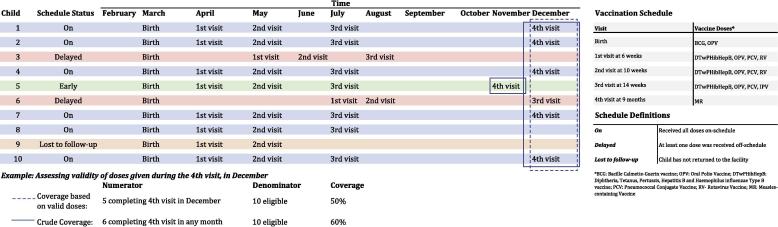
Example of calculating number of valid doses.

Validity and timeliness measured using EIR data can be based on multiple criteria and use empirical evidence that allows them to be more applicable and actionable for a particular population. For measuring vaccine validity, when using individual-level data for the numerator, children administered doses early would be considered invalid (as this is not a recommended practice), while those administered on-time or after the scheduled date would be considered valid for those doses (taking into consideration clinical guidelines). (Fig. 1) For completion of a vaccination series, validity would be dependent on whether there was the correct interval of time between all doses. The number of children receiving all doses within the appropriate intervals can then be calculated as a new measure.

**Fig. 2:**
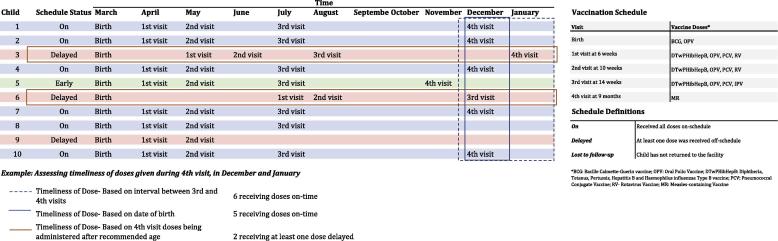
Example of differing vaccination schedules.

Once dose-validity is determined, this information can be incorporated with vaccine administration timeliness to assess and/or update a child’s vaccination schedule. With individual-level data, the timeliness of each vaccine’s administration can be assessed based on a child’s particular, age-appropriate, vaccine schedule. (Fig. 2) Vaccination timeliness can also be calculated as time since the previous dose in a vaccine series was administered. If a child falls behind on their vaccination schedule, their next scheduled visits can be updated to reflect the delay in their schedule, while maintaining the proper amount of time needed between subsequent doses to ensure acquisition of immunity. The number of children receiving a single type of vaccine on-time can be calculated along with how many received all vaccinations on-time. Large gaps in vaccination coverage have been found in some countries when comparing children receiving up-to-date versus age-appropriate vaccinations, demonstrating the added value EIRs provide by being able to actively track timeliness [24].

More defined and specific categorizations of vaccination schedules can be used when the timeliness of vaccination administration is included in performance measurements; instead of only considering if a child is up-to-date on their vaccinations, children’s vaccination schedules can be categorized as on-time (age-appropriate), delayed, or lost to follow-up (defaulter). Some organizations and countries are already routinely using these types of categorizations, such as PAHO, to evaluate missed opportunities for vaccination [22]. Because individual-level data can also track whether a child switches schedule categories, this would also need to be reflected in coverage calculations. For instance, how would a child previously lost to follow-up be included in the denominator? Additionally, tracking the proportion of doses given early would provide immunization programs with a new measure of tracking performance as it indicates whether healthcare workers are following proper immunization practices or not.

The definition of timeliness can be modified to reflect the practices of a particular population or program. For example, in Kenya, an electronic vaccine monitoring system was used to measure vaccination coverage and timeliness; “up-to-date vaccination coverage” was considered the proportion of children vaccinated by their first birthday, and “age appropriate vaccination” was considered the proportion of children vaccinated within 4 weeks of the age of vaccine eligibility. [25] Using a strict definition of timeliness, as the exact date a vaccination is due according to a child’s birth date or the administration of a previous dose, may not be recommended as this definition does not factor in the days a clinic is open or allow any flexibility in the caregiver’s schedule. Including a buffer amount of time (such as 10 days following the scheduled date) for the definition of an on-time vaccination will still allow for immunization programs to track performance and accommodate system and human factors. Additionally, this measure of timeliness can be more accommodating where children with one delayed vaccination do not necessarily need to be considered delayed for the remainder of their doses if they receive them on-time.

### 3.2 Vaccination coverage

**Fig. 3:**
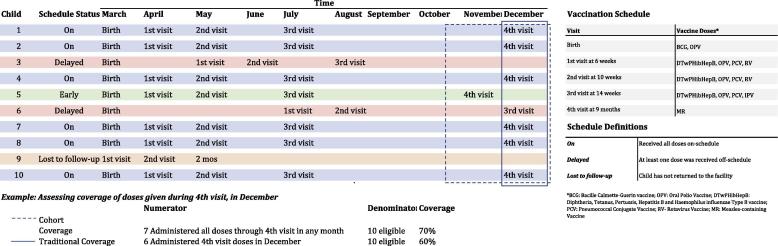
Example of cohort coverage versus traditional coverage calculations.

The use of individual-level data allows for both the numerator, number of children receiving a valid dose, and denominator, number of children recommended to receive a vaccination, to be more accurately and purposefully measured for calculating vaccination coverage. There are multiple options for calculating coverage when data are readily accessible and constantly being updated within an EIR. With the ability to include a child’s age in the vaccination coverage calculation, coverage can be calculated for a specific age cohort based on compliance with the recommended vaccine schedule [4]. Cumulative cohort coverage can be calculated on a rolling basis. (Fig. 3) The number of fully immunized children (FIC) can be calculated along with the number of children up-to-date for age. These measurements would replace the use of measles-containing vaccine coverage as a FIC proxy, which is commonly done in LMICs due to the burden of reviewing paper-based records. Coverage calculations can further be disaggregated by crude (any dose administered, regardless of timeliness) versus valid doses administered and whether children were on a delayed schedule. Use of specific time periods or age groups for the coverage denominator will be dependent on the purpose and use of the coverage estimate, each option has its own benefits and limitations for use by immunization program managers [26]. For instance, if the denominator includes a rolling cohort of children versus a fixed year or birth cohort how does this change the usefulness of the measure for a manager?

A further consideration needed for calculating vaccination coverage using individual-level data is determining which children should fall into a given target population. For some countries, target populations or catchment populations have been estimated from census data, based on the total population within a given geographic area. However, these data are often inaccurate and unspecific and therefore EIRs allow for these populations to be redefined. Coverage calculations can use the number of children registered in the EIR system or an estimate from an external source as the denominator. For instance, if a child is seen at multiple facilities, does each facility include the child in their coverage estimate or is the child only included in their “home” facility’s estimate? This question brings to light the differences in potential denominator groups; these groups can be defined by a “home” facility, the last facility a child visited, the geographic area of residence, or the most frequently visited facility. One study using IIS data to explore methods for defining denominators for vaccine coverage estimates amongst adolescents in the United States found a 20% difference in coverage between methods [27]. It is likely that the best suited definition depends on the practices of a particular population or performance measurement goals. For each of the aforementioned examples of denominator groups, the number of non-health service seeking children would also need to be accounted for in order to estimate population coverage.

### 3.3 Drop-out and lost to follow-up

Using EIR data, immunization programs can better identify children not completing their schedules and follow-up to ensure they receive their scheduled doses. The current methodology for calculating drop-out is a crude estimate as it does not take individual drop-out into consideration and compares different cohorts of children. At the aggregate level, the additional level of specificity brought by EIRs can allow immunization programs to identify customized solutions to better target these different groups, or individual children. Established vaccine registries in the United States have found that these data give supervisors more oversight over their program’s performance enabling them to take meaningful action, develop intervention strategies to target poor performance areas, improve clinic workflows, and identify opportunities for additional HCW and patient education [28].

**Fig. 4:**
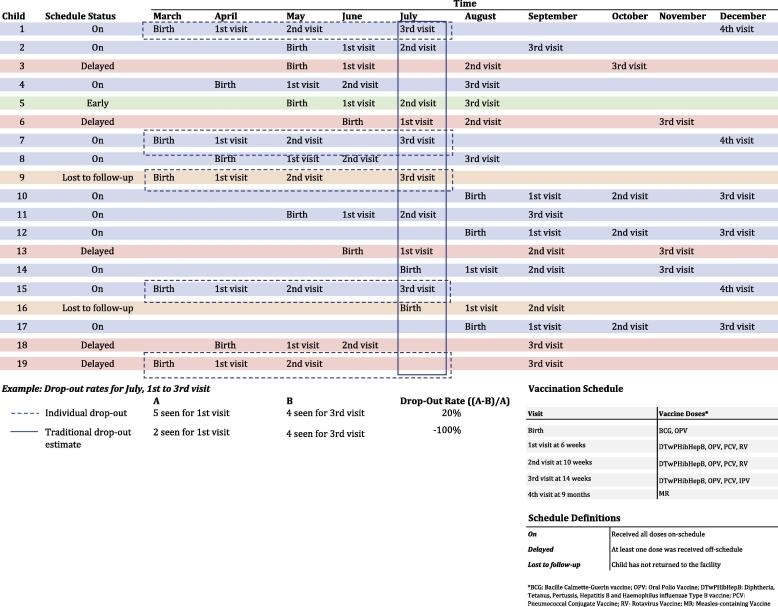
Example of individual versus traditional drop-out calculations.

Measuring the number of children lost to follow-up, or those children not returning to any facility for vaccination, can be defined using multiple criteria stemming from individual-level data. Some EIRs may allow for reasons for children not receiving a vaccine to be captured. In the case that there is a vaccine stock-out or a caregiver refuses for their child to be vaccinated, these reasons can be tracked alongside the number of children not returning for a scheduled vaccination so immunization programs can better target their activities. Individual-level data allow immunization program staff to quantify the time between visits and they can assess trends of children returning or those lost to follow-up. The definition of a child lost to follow-up can be specifically defined and made relevant to a given population based on observed trends in service delivery and healthcare seeking behaviour for that area. (Fig. 4)

Along with individual-level data, the use of new technology to capture these data increases the possibilities for better tracking children across time and geography. Centralized EIRs allow for children’s vaccination records to be linked across facilities, thereby allowing more visibility into which children are lost to follow-up versus those who have simply switched facilities, which can then help improve the accuracy of facility coverage estimates. Denominators for coverage calculations will need to capture children switching facilities over time, the impact of this movement may vary by location and size of the administrative area. With more accessible data, missed opportunities for vaccination can be assessed at sub-national levels; these are common occurrences, but are not often tracked due to the limitations of available data [29].

## 4 Challenges and considerations for using EIRs

Despite the numerous potential benefits of using data generated by EIRs, challenges and limitations with use of the data persist. LMICs transitioning from paper to electronic systems will have to consider the impact of these limitations when using EIR-generated data to calculate performance measures. Similar to many information systems, the completeness and quality of data input into the system dictates the accuracy of the estimates generated by the system, contingent on the system’s design, user compliance, and system maintenance.

Completeness of EIR datasets remains a consistent challenge for even well-established systems [30]. A study conducted in China found that discrepancies between EIR and survey data were largely influenced by under-registration in the EIR, requiring continued reliance on survey data to produce reliable vaccine coverage estimates [31]. Studies from the United States have found that electronic IISs provide more complete records when compared to medical records (paper and electronic) or parent report, but strategies are needed to improve data completeness [32]. Consistent use of unique identifiers and the utilization of systems during all immunization events is crucial for ensuring children’s records are complete. Also, deceased individuals or those moving out of the area should be designated as active/inactive to ensure that measures are not over or under inflated, as recommended in the United States [33]. Routine identification of duplicate records is also needed to ensure children or vaccine doses are not double-counted. Methods for overcoming completeness and quality issues have been studied by various immunization programs, however these methods are often tailored to health seeking practices amongst specific populations. For instance, the Oregon State IIS in the United States uses temporal comparisons to understand if vaccination coverage is better or worse than expected to mitigate issues of data incompleteness [34], [35].

Potentially significant data cleaning may be needed to calculate redefined vaccination measures using electronic systems if built-in data quality checks and verification are not robust or if users are poorly trained on preventing these types of issues. Programs transitioning from paper to electronic systems may grapple with new data quality issues that will require stringent guidance on how to properly input vaccination history data. For instance, if a child has a documented 3rd dose in a vaccination series recorded in their home-based record, should a nurse assume they received the 1st and 2nd dose as well? If legacy data is not back-entered consistently for all children, vaccination coverage estimates would be inaccurate for all doses administered prior to the introduction of the EIR. The American Immunization Registry Association (AIRA) has recently published guidance on data quality practices for use of EIR data to tackle these potential limitations [36].

Calculating accurate estimates of performance measures using EIR data will likely remain elusive until the challenges mentioned above have been addressed by immunization programs. As transitioning countries continue to use paper based tools, along with electronic systems and surveys, they will have to determine which data source produces the most valid and accurate estimates. One study in Kenya found that routine survey data underestimated vaccine coverage while administrative data overestimated coverage, compared to EIR data [25]. If the estimates generated by each data source are considered valid in their own respect, how will program managers determine which estimate is accurate for a particular population? Additionally, accurately estimating the target population is likely to remain a challenge as the use of individual-level data made accessible by EIRs can only help estimate vaccination coverage among the healthcare seeking population captured in the system. As countries transition from paper to electronic data systems, they will have to decide when the EIR data is considered accurate enough to replace paper-based data and satisfy reporting requirements of WHO and Gavi, the Vaccine Alliance.

Additionally, although the effectiveness of electronic systems for improving immunization program performance seems promising, evidence gaps exist [37], [38]. On-going health system and government limitations also threaten the sustainability of the EIRs. [39], [40], [41] In PAHO, countries implementing EIRs have found that it is a time-intensive process that requires continuous human and financial support [16]. Challenges to using EIR data can be exacerbated if the system is not implemented in a well-functioning environment that provides consistent system maintenance and supports data use.

## 5 Future directions

Future upgrades to EIRs and IISs can include clinical decision support features to help HCWs determine valid doses and to track patient contraindications and precautions for administering specific vaccines to increase patient safety. Dose validity can further be refined as additional information is incorporated into an immunization information system, e.g., history of immunity or disease, vaccine product details, route of administration, and cold chain history. Pairing individual-level data with other health facility measures and patient characteristics introduces new possibilities for assessing program performance. For instance, stock data can be compared to the individual data on vaccines administered and scheduled to more accurately estimate vaccine supply and demand at the facility level, improving vaccine ordering practices. As technology continues to improve, more sophisticated software functionality can feed the data into algorithms and predictive modelling to help HCWs identify children at risk for not returning for a scheduled vaccination.

Although EIR data has been most commonly used to evaluate vaccination coverage, the data may prove valuable to other programs and activities beyond routine immunization [42]. The data can be used to identify immunity among children during a VPD outbreak response to quickly vaccinate only those susceptible, allowing for resources to be conserved. Vaccine efficacy studies can utilize individual-level data for new vaccine introductions. Individual RHIS data can be utilized for program evaluations and research studies using quasi-experimental designs [43]. As one of the strongest childhood health programs, better understanding vaccination coverage using redefined definitions can help improve countries’ push towards healthcare equity and universal health coverage, by helping to identify children left out of healthcare services. When designing or updating national electronic IISs, countries should consider which measures they are most interested in tracking over time a priori and build these into their systems with considerations for how they can be used by other programs [14].

## 6 Conclusion

As the use of EIRs continues to increase, countries will have the opportunity to collect more timely, accurate, and particular information on childhood vaccination coverage, but will continue to face limitations for measuring progress. Using individual-level data to redefine and create new common measures of immunization program performance provides numerous opportunities for supervisors and managers to have better oversight and improved decision-making capacity for their activities. The challenges of data quality and completeness will continue to be problematic until EIRs are consistently used for all children within a geographic area. However, these data are still an improvement on historic methods for measurement. Countries that are close to reaching target vaccination coverage goals can utilize the particularity of measures based on individual-level data to pinpoint their weakest areas and use resources efficiently to improve coverage against VPDs.

## Funding

This work was supported by the Bill & Melinda Gates Foundation, Seattle, WA. [grant number OPP1042273].
